# Reduction of Multipath Effect in GNSS Positioning by Applying Pseudorange Acceleration as Weight

**DOI:** 10.3390/s24216880

**Published:** 2024-10-26

**Authors:** Kwan-Dong Park, Woong-Jun Yoon, Jong-Sung Lee

**Affiliations:** 1 Department of Geoinformatic Engineering, Inha University, 100 Inha-ro, Incheon 22212, Republic of Korea; yoongoodid@naver.com (W.-J.Y.); jslee@gps2u.kr (J.-S.L.); 2 PP-Solution Inc., 606 Seobusaet-gil #B-2311, Seoul 08504, Republic of Korea

**Keywords:** GNSS, multipath, range acceleration, signal-to-noise ratio (SNR), weight model

## Abstract

A novel approach is proposed to mitigate the multipath effect that is considered a major source of error in global navigation satellite system (GNSS) positioning in urban areas. We utilize code pseudorange acceleration measurements as a weight in a least squares estimation process. If GNSS signals are reflected off a surrounding surface, they cause large variations in the recorded pseudorange measurement. Accelerations computed at each epoch with three consecutive pseudoranges exhibit significant fluctuations in a multipath signal. As a result, positioning accuracy improved by 75% horizontally and 79% vertically compared to not applying any weight. Even when multipath errors exist, the range acceleration (RA) value is sometimes low at many epochs. When a threshold value for the signal-to-noise ratio was additionally applied besides RA, the positioning accuracy at two test sites (including a deep urban environment) improved by more than 80% in both horizontal and vertical directions.

## 1. Introduction

In recent times, the ability to obtain accurate and real-time positioning has become increasingly important in various fields such as autonomous vehicles, unmanned aerial vehicles, and location-based services (LBSs). As demand for higher quality and stability in localization grows, different sensors are utilized. However, there are limitations to image-based positioning using cameras, especially in situations where there are no established image reference point infrastructures or precise electronic maps. Furthermore, positioning using radar and lidar will measure the relative positions of surrounding objects but cannot determine the absolute position of an object. Therefore, the main methods for acquiring an absolute position are satellite navigation and communication network radio frequency (RF) positioning. Satellite navigation, provided as a global service by America, Russia, China, and Europe, is a key form of RF positioning. This service, offered by the global navigation satellite system (GNSS), is known as GNSS positioning. Although GNSS positioning typically has an error of several meters with low-cost modules, the multipath effect in urban environments with dense buildings can cause errors of up to hundreds of meters or more, making multipath error a significant limitation.

Multipath error in GPS positioning is caused by the satellite signal reflecting off the terrain or buildings near the antenna. It occurs frequently in urban areas with dense buildings and when satellites have low elevation angles. Unlike ionospheric and tropospheric delay, multipath error cannot be eliminated by relative positioning [1,2], and it is difficult to predict. Therefore, research has been conducted for decades on reducing the effects of multipath error [3,4,5,6]. The two notable literature survey-type papers review technological developments and advances in reducing the adverse multipath effect during the past 30 years [2,7]. Most recently, attempts to adapt machine learning and deep learning approaches to solve the multipath problem encountered in urban canyons [7,8] and to tackle the multipath effect in processing the raw GNSS measurements collected by smartphones [9] are underway. Solutions to multipath errors can be placed into two categories; the first is receiver and antenna improvement and the second is software-based. Software approaches can again be divided into two operation modes, post-processing and real-time processing.

The objective of this study is to find robust solutions in real-time GNSS navigation. Although many studies have been conducted, there are some limitations in applying the strategy to real-time positioning. Recent attempts have been made to reduce multipath error by using the signal-to-noise ratio (SNR) and elevation angle as weights. Li and Wu [10] proposed a weight model to lessen adverse multipath effects in urban environments by modeling the SNR as an exponential function and exploiting the fact that the SNR decreases when GPS signals reflect off buildings. Tay and Marais [11] proposed a weighting model that uses both elevation angle and the SNR based on the fact that low-altitude satellites are relatively more affected by buildings. Kubo et al. [12] did reduce positioning errors by considering the CN0 magnitude and its variability characteristic in the deep urban environment. Although the exponential function SNR model and the model combining elevation angle with the SNR are somewhat effective, they can still cause a positioning error of more than 10 m in urban environments.

This paper presents a method for identifying multipath errors by analyzing the highly variable characteristics of pseudorange observations in order to improve the accuracy of GNSS positioning in urban areas. We devised a new strategy to enhance positioning accuracy by giving less weight to observations affected by multipath errors. The effectiveness of our multipath error reduction model was verified at two field sites. Section 2 of this paper details pseudorange characteristics and the first and second time derivatives in cases where multipath errors are caused by a single reflector. In Section 2.4, we demonstrate that positioning performance is enhanced by using our model. Section 3 outlines a strategy for fine-tuning the model and incorporating an additional SNR threshold. Lastly, Section 4 presents a discussion of the results obtained from the field tests.

## 2. Range, Range Rate, and Range Acceleration

### 2.1. Test Setup and Data Collection

**Figure 1:**
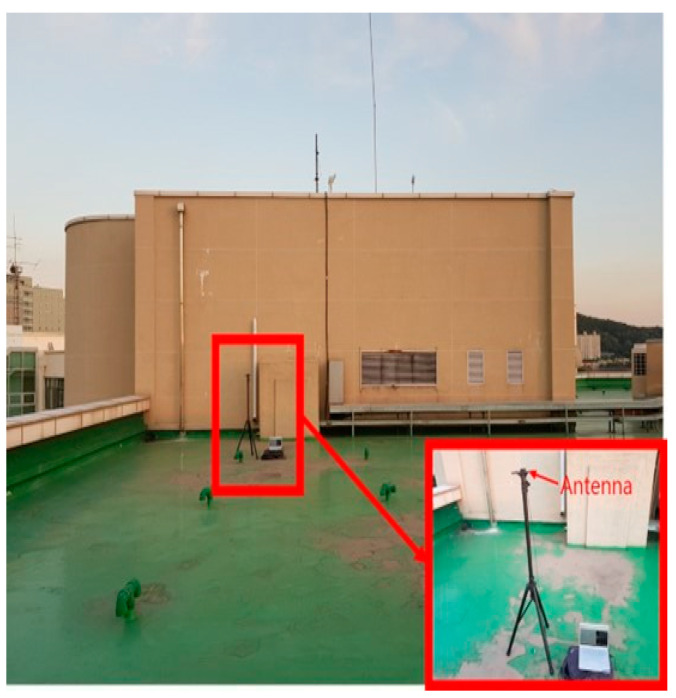
Except for the elevator room to the south, the test equipment set up on the Inha University building roof can be considered an open-sky environment.

Before analyzing multipath error characteristics due to multiple surfaces, the multipath effect caused by a single reflecting structure was investigated. For this purpose, the rooftop of a building (an open-sky environment) shown in Figure 1 was selected. The only structure on the rooftop is an elevator mechanical room, and a tripod was set up in front of it. A patch antenna was placed on the tripod, and the data were recorded with a laptop. The receiver was a low-cost single-frequency u-blox EVK-M8T. Except for the elevator room shown in the picture, no structure blocked the signal in any other direction, so the effect caused by a single reflector could be accurately identified.

**Figure 2:**
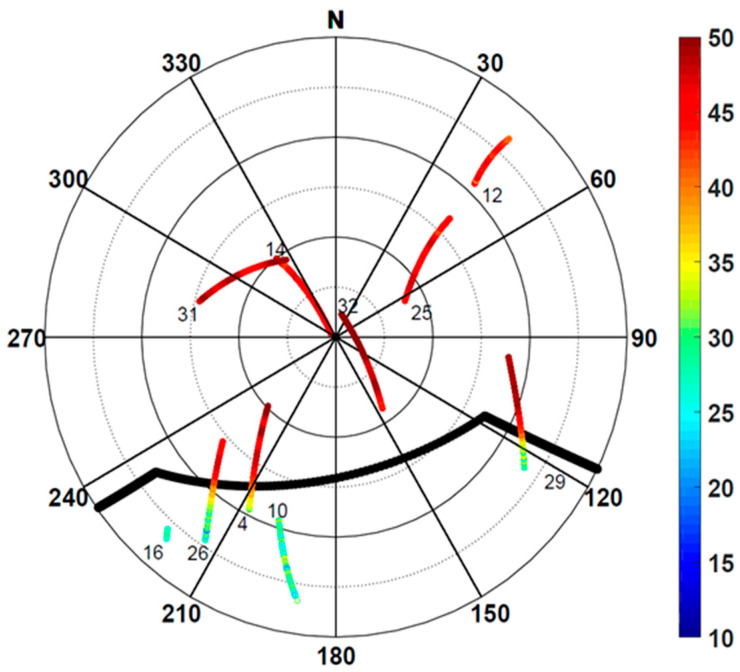
This skyplot shows tracks of 10 satellites observed during the test period with signal strengths in dB denoted as a color bar on the right-hand side. The satellite PRN number is included at the starting point of each track. The thick black solid line denotes the signal obstruction from the elevator mechanical room to the south.

The data were collected for 8 h and 25 min (from 06:35 to 15:00 UTC) on 24 January 2018 at one-second intervals. Figure 2 is a skyplot of the satellite signals received during the 60 min from 06:36 to 07:36. Each PRN number on the skyplot corresponds to the first observation point. SNR values are depicted in color as defined on the color bar. A total of 10 satellites were observed and as expected, satellites with high elevation angles showed larger SNR values.

In Figure 2, the thick solid black line is the outline of the elevator mechanical room shown in Figure 1, and the outline coordinates were obtained through RTK surveying. The room occupies azimuth angles from 120° to 240° and blocks signals with an elevation angle of less than 45° in the azimuth angle range of 150°–180°. There were five satellites with a line of sight (LoS) not secured due to obstruction, namely PRNs 4, 10, 16, 26, and 29. Among them, PRNs 4, 26, and 29 pass through the outline. These three satellites show low SNR values inside the outline, i.e., the section where the LoS is not secured. Then, the SNR increases rapidly as the signal leaves the boundary. Therefore, the signal has a low SNR due to multipath error in the area where the LoS is not secured and shows a typical magnitude of SNR values when there is no obstruction.

### 2.2. Range vs. Range Rate vs. Range Acceleration

**Figure 3:**
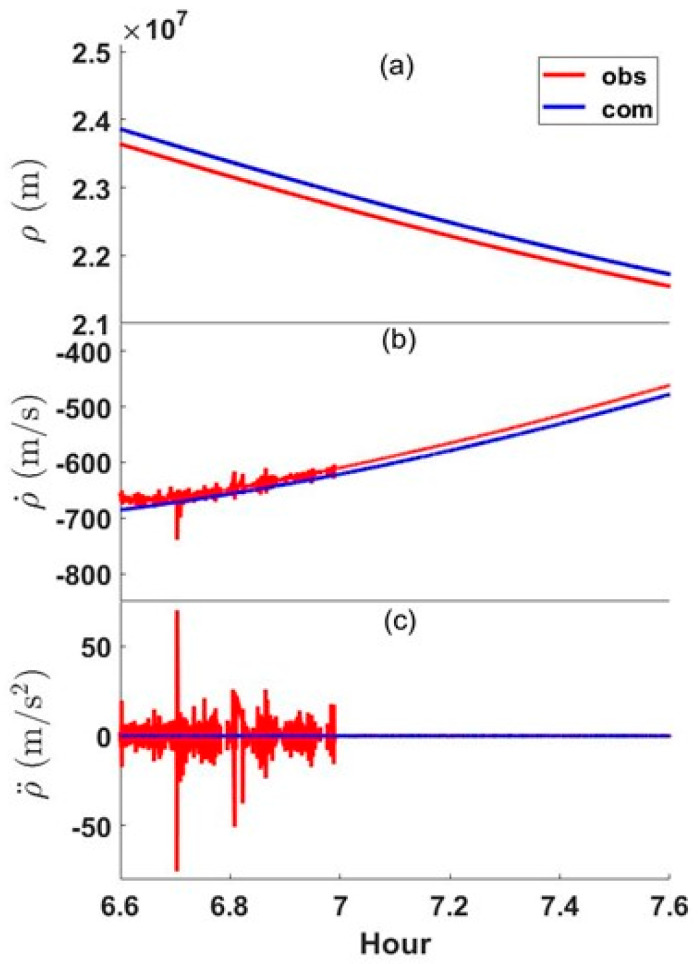
Measurements and computations for pseudorange ρ, range rate $\dot{\rho }$, and range acceleration $\ddot{\rho }$ of PRN 26. (**a**) Pseudorange ρ, (**b**) range rate $\dot{\rho }$, (**c**) range acceleration $\ddot{\rho }$. Red and blue lines denote observed (obs) and computed (com) values, respectively.

We used the dataset described in Section 2.1 to derive the range rate (RR) and range acceleration (RA). RR is the time rate of change for the pseudorange measurement, whereas RA corresponds to the second time derivative of the range. PRN 26 in Figure 2 was observed from 06:35 to 13:06 UTC, and the pseudorange, RR, and RA measurements from 06:36 to 07:36 are depicted in Figure 3. The upper part of the figure shows two pseudorange values; red is the observed pseudorange recorded at the receiver, whereas blue is the computed distance from the receiver to the satellite by predicting the satellite coordinates from the broadcast ephemeris.

Seen in Figure 3a is the bias between the observed pseudorange and the calculated value; the size of the bias starts at 22,254 m and gradually decreases until it reaches 17,811 m. When converted to time, this corresponds to approximately 0.742 ms and 0.594 ms. Several types of error may have affected this bias, but the main cause is that clock errors are not modeled. In calculating the pseudoranges in Figure 3, the satellite clock offset was modeled. Thus, it is assumed that receiver clock offsets, besides unmodeled tropospheric and ionospheric errors, affect the bias. However, bias analysis is unnecessary in this study and was not considered further. The reason should be clear in the subsequent descriptions of RR and RA computations.

Figure 3b shows the RR, which is the time change rate of the pseudorange. Since GPS satellites orbit the Earth’s center of mass and the observation point is located on the surface of the Earth, the distance between the receiver and satellite is not constant. Therefore, the time change rate of the pseudorange cannot be zero and its magnitude changes smoothly within a range of approximately ±800 m/s. One can see that PRN 26 is a rising satellite from the skyplot. Thus, the pseudorange value continuously decreased after the first observation, and its value also appears to be within ±800 m/s (from −700 to −200). As with the computed pseudorange, the ${\dot{\rho }}_{com}$ values (RRs calculated based on the receiver’s coordinates and the satellite’s orbital information) change very smoothly. On the other hand, RR values calculated from the data recorded at the receiver (${\dot{\rho }}_{obs}$) were quite noisy from 06:36 to 07:00 when the LoS was not secured due to the elevator room. It was determined that the corresponding portion of the data was affected by the multipath effect. Because the signal was recorded at the receiver during the obstructed period, it must have been received indirectly by diffraction or reflection (in other words, under the multipath effect). We found some missing epochs believed to be caused by excessive signal interference.

The instability of the RR in Figure 3b and the corresponding index can be used as a weight in the GNSS positioning algorithm. However, it is difficult to decide a specific number for the multipath classification criterion because the RR value depends on the satellite position and the direction of motion, even though it stays within the range from −800 to +800 m/s. What is needed for stable satellite navigation is a reasonable threshold and thus, RR cannot be a suitable candidate. For this reason, we tried the second time derivative of range, or range acceleration, which is the RR time-differentiated again. Figure 3c shows RA computations, where the calculated RA value (in blue) is almost always concentrated on the line $\ddot{\rho }$ = 0 m/s^2^. On the other hand, the RA values derived from recorded observations (${\ddot{\rho }}_{obs}$, in red) are close to zero from 07:00 to 07:36 when the LoS is secured. However, RAs rapidly fluctuated with maximum values of ±75 m/s^2^ when affected by the multipath effect.

**Figure 4:**
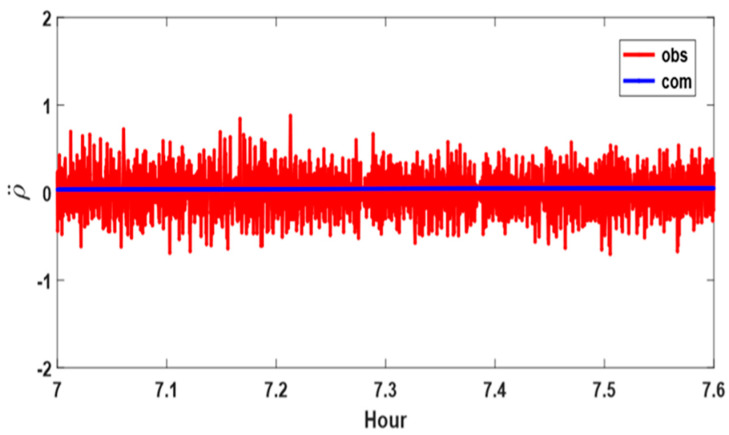
Range acceleration measurements and computations for PRN 26 while the test antenna maintained a clear line of sight to the satellite. Red and blue lines denote observed (obs) and computed (com) values, respectively.

The RA time series after 07:00, when PRN 26 was completely free from the influence of the building, is shown in Figure 4, with the vertical axis limited to ±2. This period corresponds to the situation where the LoS is perfectly secured. In Figure 4, observe that ${\ddot{\rho }}_{com}$ consistently remains along the line $\ddot{\rho }$ = 0 m/s^2^. On the other hand, ${\ddot{\rho }}_{obs}$ reached a maximum value of almost 1.0 m/s^2^, with an average value of 0.21 m/s^2^ and standard deviation of 0.15 m/s^2^. In conclusion, since the RA characteristics are clearly distinguished, depending on the presence or absence of multipath error, it can be used as an index for a weight model in data processing.

### 2.3. Range Acceleration Computations

**Figure 5:**
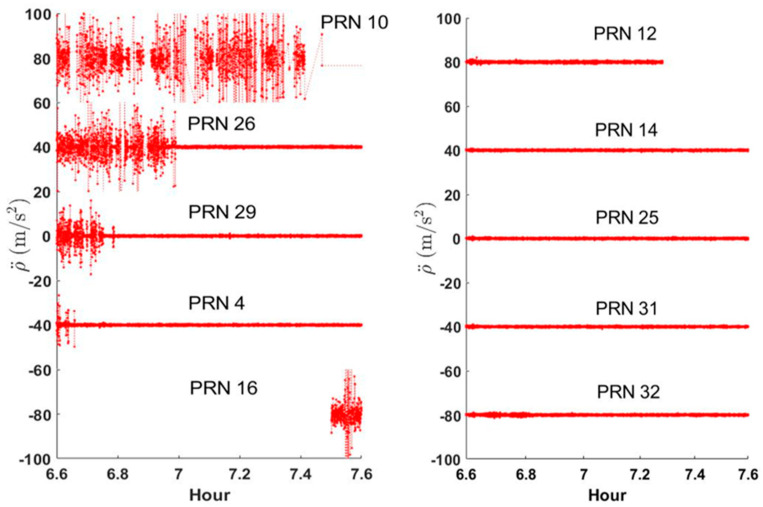
Range acceleration measurements for all 10 satellites observed during the test. Five plots on the left-hand side show satellites affected by signal obstruction (the multipath effect), while five plots on the right-hand side maintained clear LoS vectors. Except for PRN 29 and 25, vertical offsets were applied for clear visual interpretation.

While the RA value of PRN 26 was close to zero when the LoS was secured, it fluctuated significantly when multipath errors occurred due to the reflector. As one can see from the skyplot in Figure 2, visibility to satellites 4, 10, 16, and 29 was also obstructed. Therefore, we examined whether a phenomenon similar to the RA variation characteristic of PRN 26 also occurred for the four other satellites. The result is shown on the left side of Figure 5. Looking at the temporal variation, the LoS vector to PRN 10 cannot be secured most of the time, showing the largest variations for the longest time. PRN 16, shown at the bottom of the left side of Figure 5, exhibited relatively large variations because it was occluded throughout the analysis period.

For PRNs 26, 29, and 4, which were obscured by the elevator room and then cleared of the obstruction, RA maintained a stable value near zero after leaving the influence of the building. The right-hand side of Figure 5 shows five satellites unaffected by the multipath interference. In contrast to the satellites on the left side, they show RA values close to zero all the time. The mean and standard deviation for ${\ddot{\rho }}_{obs}$ from those five satellites are, from top to bottom, 0.24 ± 0.19, 0.16 ± 0.14, 0.19 ± 0.15, 0.18 ± 0.14, and 0.24 ± 0.19 m/s^2^. The mean values are all greater than zero and the maximum value was 0.24 m/s^2^. Each standard deviation was less than 0.2 m/s^2^. The level of noise in the RA observations for the 10 satellites is closely related to the presence or absence of signal obstruction. This is a very consistent result, validating our choice of RA as a feasible multipath effect indicator.

In an open-sky situation, RA changes smoothly, with most values close to zero. Contrarily, in a multipath-inducing environment, RA has severe noise. To use this characteristic as weight in positioning, the difference between the calculated RA and the observed RA can be used. That is, difference $∆\ddot{\rho }$ (${\ddot{\rho }}_{obs}-$ ${\ddot{\rho }}_{com}$) is a type of residual. Thus, if the difference is large, one can consider the corresponding signal to be the multipath signal. To use $∆\ddot{\rho }$ as a weight for data processing, ${\ddot{\rho }}_{com}$ must be calculated, and receiver coordinates are required. However, the reliability of the coordinates estimated in places where the observation environment is not good is bound to be low. Therefore, another index was devised to replace $∆\ddot{\rho }$.

As confirmed in Figure 3 and Figure 4, the calculated RA value ${\ddot{\rho }}_{com}$ is very close to zero and more stable than the observed RA value ${\ddot{\rho }}_{obs}$. To determine the range of ${\ddot{\rho }}_{com}$ values, RA was calculated for all 26 satellites observed at the Jinju Permanent GNSS station in Korea for the 12 h from 02:00 to 14:00 on 30 August 2017. The maximum value for the 26 satellites was approximately 0.081 m/s^2^. This proves that the fluctuation in ${\ddot{\rho }}_{com}$ is small compared to ${\ddot{\rho }}_{obs}$, which shows a maximum of ±50 m/s^2^ in Figure 3. Thus, ${\ddot{\rho }}_{com}$ is judged to have a negligible effect when applied as a weight to the positioning algorithm. Therefore, when range acceleration is used as a weight in the positioning algorithm, one can use ${\ddot{\rho }}_{obs}$ only, excluding ${\ddot{\rho }}_{com}$ from $∆\ddot{\rho }$. In other words, the weight can be determined only with the pseudorange observation value recorded at the receiver, regardless of the receiver position. In the remainder of this paper, ${\ddot{\rho }}_{obs}$ is represented as $\ddot{\rho }$ for simplicity.

So far, we have only conducted RA analysis for a stationary site. To simulate a moving platform, we assumed a case where the receiver moved northward from the Jinju site at 100 km/h and calculated ${\ddot{\rho }}_{com}$. The absolute value showed a maximum of 0.085 m/s^2^ when all 26 observed satellites were considered. Therefore, we can conclude that ignoring ${\ddot{\rho }}_{com}$ should not pose a problem, even when moving at a high speed.

### 2.4. Range Acceleration as a Weight in Data Processing

For positioning accuracy analysis, we tested three noise models, namely a linear, polynomial, and exponential model denoted as follows:

(1) $$\sigma_{i}=\alpha \cdot {\ddot{\rho }}_{i},$$

(2) $$\sigma_{i}=\alpha \cdot {\ddot{\rho }}_{i}^{k},$$

(3) $$\sigma_{i}=\alpha \cdot e^{k\cdot {\ddot{\rho }}_{i}}$$

**Figure 6:**
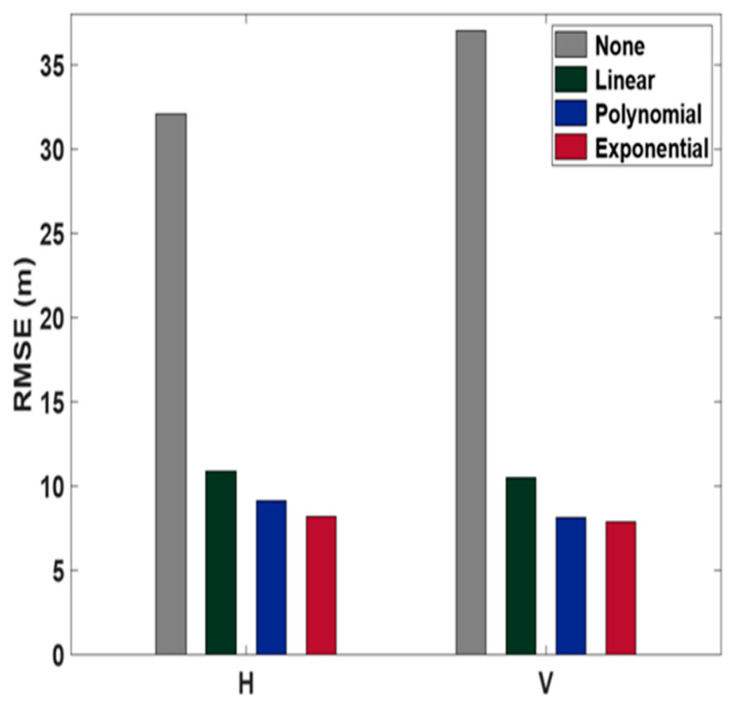
Horizontal (H) and vertical (V) RMSE values from three different weighting models compared with no range acceleration.

Each model incorporates a variance term, $\sigma_{i}$, for the measurements, with optimized parameters α and *k* determined empirically. The unit for $\ddot{\rho }$ is meters per second squared. The C/A code pseudoranges collected at one-second intervals for one hour (from 06:36 to 07:36) were used. Least squares estimation was applied to compute the receiver coordinates and the clock offset, totaling four unknowns. The true coordinates of the antenna from Figure 1 were obtained through RTK surveying and used as the reference for position accuracy computations. Root mean square error (RMSE) values for the entire hour are displayed in Figure 6.

When no weight was applied, positioning accuracy in terms of the RMSE was 32.1 m horizontally and 37.0 m vertically. When using the linear model, accuracy improved by approximately 65% to 10.9 m horizontally and 10.5 m vertically compared to no weight applied. When the quadratic polynomial model was used, accuracy improved by approximately 70% to 9.1 m horizontally and 8.1 m vertically, surpassing the results from the linear model. Finally, with the exponential function, accuracy was 8.2 m horizontally and 7.9 m vertically, with improvement rates of 75% and 79%, respectively, compared to no weight applied. Therefore, we can conclude that the exponential function is the most effective choice from among the three models.

## 3. Improvement in RA Weight Model

**Figure 7:**
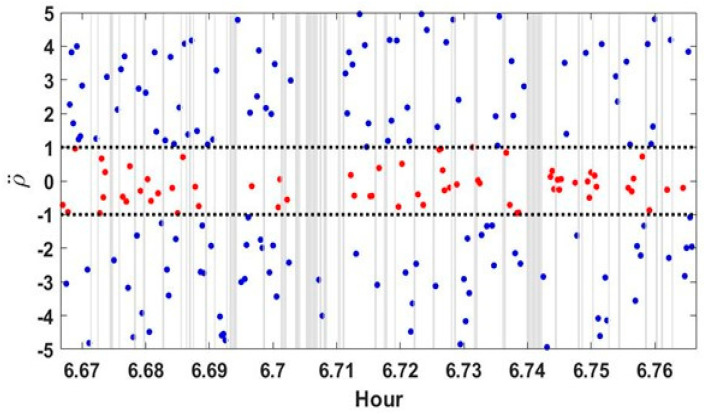
Low pseudorange acceleration values from multipath interference. Values within ±1 m/s^2^ are denoted by red dots.

The multipath error index adopted in this study, RA (or $\ddot{\rho }$), has a value within approximately ±1 m/s^2^ for a direct signal but showed large fluctuations when affected by multipath errors. Using this characteristic, a new multipath effect reduction model was developed and we confirmed that it improved positioning accuracy. However, we discovered some cases where adverse effects show up. The reason was that the computed $\ddot{\rho }$ can have unexpectedly low values, even at epochs where the multipath effect can be confirmed. Even when affected by multipath errors, there were epochs at which the fluctuation of $\ddot{\rho }$ was very low, and these values could not be distinguished from direct LoS signals (Figure 7). When a low $\ddot{\rho }$ value was obtained from an instance of multipath interference, it was confirmed that accuracy significantly lessened at the epoch when the calculated RA was applied to the weight as is.

An additional multipath index was designed to countermeasure the adverse effect of low $\ddot{\rho }$ values obtained from the multipath signal. The signal strength recorded at the receiver, the SNR, has been proposed by many researchers as a criterion for determining the presence of a multipath signal [10,11,13,14]. As can be observed from a visual inspection of Figure 2, the SNR value changes rapidly near the boundary. SNR measurements vary based on satellite elevation angle, antenna characteristics, and receiver hardware configuration. Because the same receiver and antenna were used, only the SNR variations due to the elevation angle were considered in this study.

**Figure 8:**
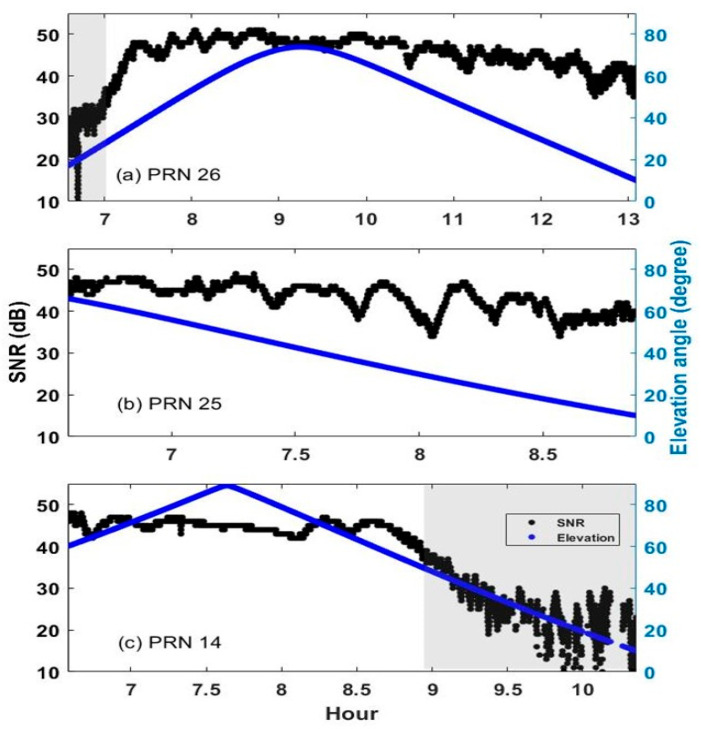
The SNR and elevation angle for three GPS satellites. Gray areas correspond to periods when the satellite was not within visible range and signals were affected by the multipath effect.

Figure 8 shows the elevation angle and SNR for PRNs 14, 25, and 26, where the elevation cutoff angle was taken at 10°. This is because the troposphere and ionosphere have significant effects below the cutoff angle. The gray areas in the charts are where the satellite signal was obstructed by the building and the LoS was not completely secured. The elevation angle of satellite PRN 26 showed a maximum of 75° at 09:12 and a minimum of 10° at 13:00. The SNR was about 50 at an elevation angle of 75° and about 40 at an elevation angle of 10°. It should be noted that the SNR showed a low value of about 30 in the gray area before 07:00 but then increased rapidly as it approached the boundary of the building. PRN 25 was not affected by the building, so the LoS was secured consistently. At a maximum elevation angle of 67°, the SNR was 47 and even at the lowest elevation angle of 10°, it showed a relatively high SNR of 40. Similar to PRN 26, the elevation angle and SNR of satellite PRN 14 showed a low SNR from around 09:00, when it began to be affected by multipath interference. Analysis of the SNR characteristic showed that elevation angle had an impact on the SNR, but it remained above 35 when there was no interference from buildings. However, it decreased rapidly, regardless of the elevation angle, when influenced by a building obstruction. Similar phenomena were found in satellites other than the three shown.

**Figure 9:**
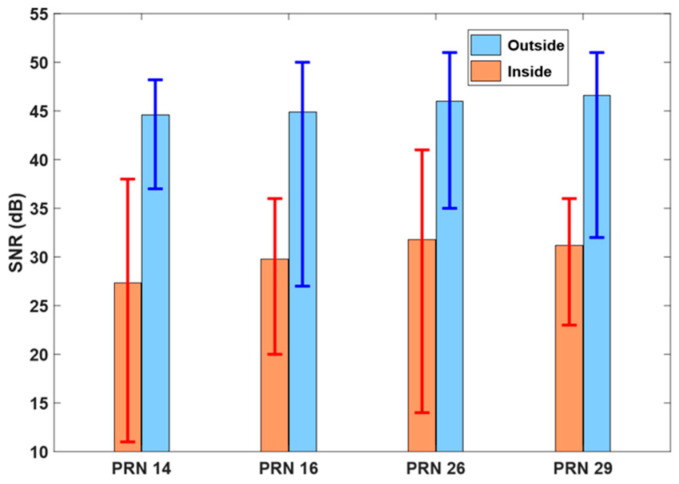
SNR observations inside and outside epochs where the satellite cannot obtain a clear LoS vector. Bar height shows the average SNR value; error bars denote minimum and maximum SNR.

When the LoS is secured, the SNR value does not decrease drastically even if the elevation angle is low. However, the SNR decreases significantly where interference by the building is suspected. Figure 9 shows the statistics obtained from four satellites affected by the building. The average SNR was 27 to 32 when affected by the building, and the range was 45 to 47 when there was no multipath influence. We established a strategy to set a specific SNR value as the ceiling and classified anything below the threshold as affected by multipath interference. This criterion is to assign the weight model $\sigma_{i}=\alpha \cdot e^{k\cdot {\ddot{\rho }}_{i}}$ based on the RA value first and then apply the SNR threshold. Through various tests, we confirmed that setting the threshold to 40 and fixing the RA value to 100 m/s^2^ if the SNR is lower than 40 is very effective.

**Figure 10:**
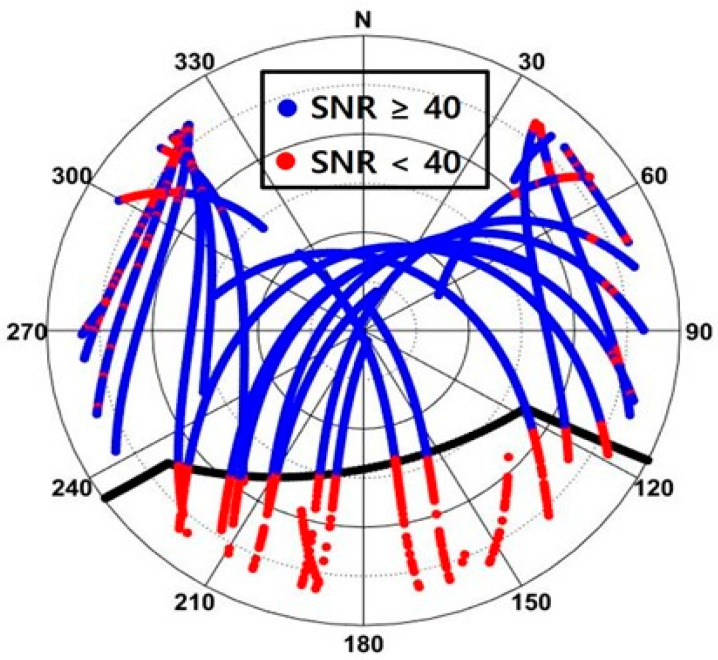
Abrupt changes in the SNR as observed across the building boundary (the black solid line).

Figure 10 is a skyplot for all satellites observed for approximately eight-and-a-half hours from 06:35 to 15:00, with SNR values above 40 indicated in blue and values below 40 indicated in red. The outline of the elevator room (the solid black line) closely aligns with the boundary between blue and red. Thus, it can be concluded that the choice of 40 is reasonable. Meanwhile, because the test antenna experiences the multipath effect from other distant buildings, red dots appear within the azimuth range of 300°–320° plus some spots outside the boundary.

## 4. Results and Discussion

The RA-based weight model and the SNR ceiling adaptation strategy were tested and validated at two static sites. First, data collected from a building rooftop were analyzed, and then a typical urban site was selected. In the urban environment especially, there are several high-rise buildings, so one can expect an abundance of multipath signals.

### 4.1. The Building Roof

**Figure 11:**
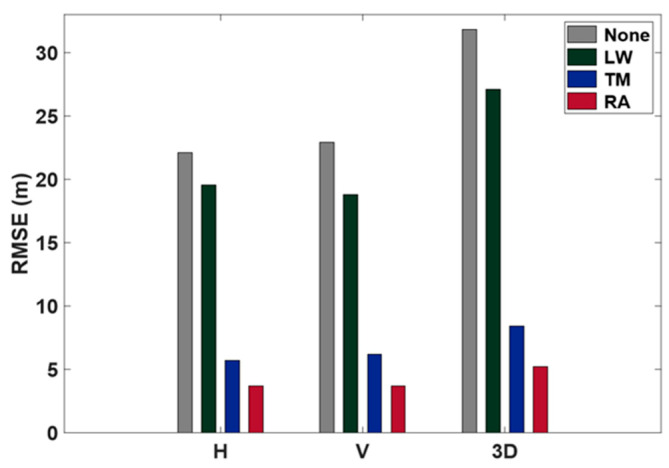
Building roof test: RMSE from three different weighting models compared to no weight applied. LW denotes the Li and Wu [10] model; TM denotes the Tay and Marais [11] model; RA denotes the model from this study based on pseudorange acceleration.

“Building Roof” refers to an environment with a single reflector in an open area, as shown in Figure 1. Data were collected from the building roof every second from 08:00 to 09:00 UTC on 19 September 2018 using the u-blox EVK-M8T receiver and the small active antenna from the evaluation kit. The data were processed using the newly developed model, and the results were compared with those obtained by models developed by Li and Wu [10] and Tay and Marais [11]. Hereafter, the newly developed model is referred to as RA, the Li and Wu [10] model as LW, and the Tay and Marais [11] model as TM. Estimated coordinates were compared with the true coordinates. The horizontal and vertical positioning accuracy for each model is presented in Figure 11 as a bar graph.

Figure 11 shows that both models improved accuracy, with the TM model being more accurate than the LW model. LW model positioning resulted in an RMSE of 19.5 m horizontally, 18.8 m vertically, and 27.1 m in 3D. In comparison, the TM model showed an RMSE of 5.7 m horizontally, 6.2 m vertically, and 8.4 m in 3D. When no weight was applied, the RMSE was 22.1 m horizontally, 22.9 m vertically, and 31.8 m in 3D.

The RA model developed in this study improved accuracy by 83% horizontally, 84% vertically, and 84% in 3D. It had an RMSE of 3.7 m horizontally, 3.7 m vertically, and 5.2 m in 3D. This improvement was greater than both the LW model (12% horizontally, 18% vertically, and 15% in 3D) and the TM model (74% horizontally, 73% vertically, and 74% in 3D), proving the effectiveness of the RA model in reducing multipath errors.

### 4.2. Urban Canyon

**Figure 12:**
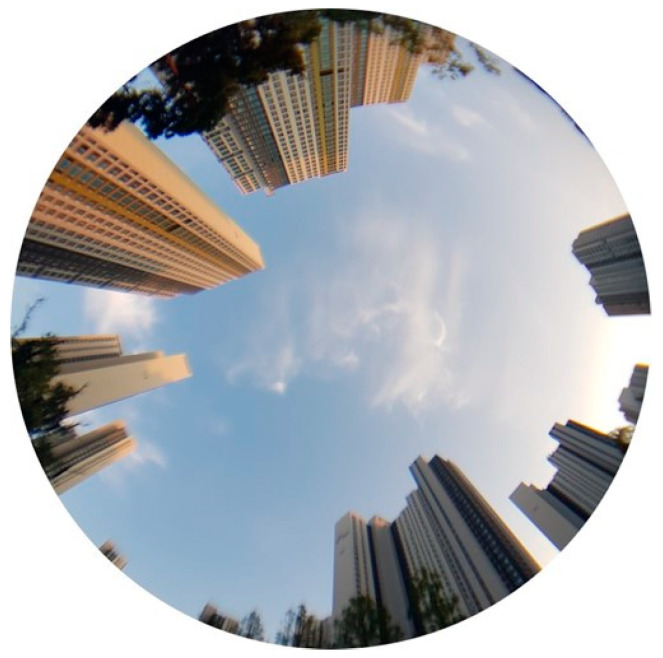
A wide-angle view from the urban canyon test site. The boundary of the circle corresponds to an elevation angle of 20°.

To test the performance of the RA model in an urban setting, we collected measurements in an area with tall buildings. Data were collected every second for 30 min from 04:26 to 04:56 UTC on 19 September 2018. We used the EVK-M8T receiver from u-blox, similar to the setup used on the building roof. Figure 12 is a photo of the site with 40-story buildings in most directions. The outer edge of the photo corresponds to an elevation angle of 20°. It was challenging to secure four or more direct LoS GPS satellites due to the poor reception environment. Therefore, we included BeiDou observations along with GPS to increase the number of navigation satellites. By integrating BeiDou measurements, we had at least 13 visible satellites, which ensured stable data processing. We obtained true values for the receiver coordinates using RTK surveying, and the positioning results were validated by repeating the procedure in the previous subsection.

**Figure 13:**
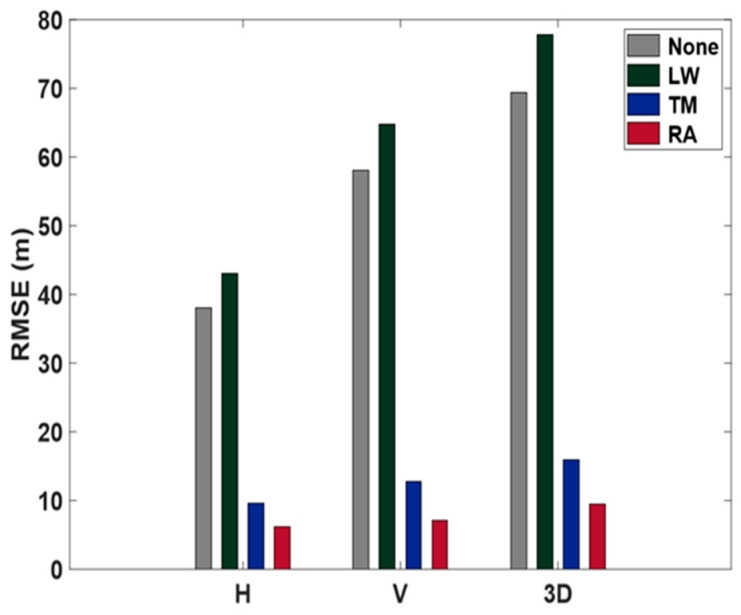
Urban canyon test: RMSE from three different weighting models compared with no weight applied. LW denotes the Li and Wu [10] model; TM denotes the Tay and Marais [11] model; RA denotes the model developed in this study.

Figure 13 shows the performance of each model. When no weights were applied, the horizontal RMSE was 38.0 m, the vertical RMSE was 58.0 m, and the 3D RMSE was 69.3 m. This indicated a significant positioning error due to multipath signals from the surrounding buildings. After applying the LW weight model, RMSE values were 43.1 m horizontally, 64.8 m vertically, and 77.8 m in 3D. The TM model demonstrated improved accuracy, with RMSE values of 9.6 m horizontally, 12.7 m vertically, and 16.0 m in 3D. It is important to note that the LW model led to accuracy degradation compared to the no-weight case. This degradation in performance is attributed to variations in SNR characteristics across different receivers, highlighting the need for receiver-specific optimization.

The TM model showed an accuracy improvement of 75% horizontally, 78% vertically, and 77% in 3D. The accuracy of our own developed model was 6.2 m horizontally, 7.1 m vertically, and 9.4 m in 3D, a horizontal improvement of 83%, 88% vertical, and 86% in 3D. The positioning results in the urban canyon environment showed that the developed model showed high accuracy improvement (over 80%) even in the urban canyon. Compared to the TM model, accuracy improved by 35% horizontally, 44% vertically, and 41% in 3D. This indicates that the RA model using the code pseudorange measurement is more effective in reducing multipath errors than existing models using the SNR, elevation angle, etc., even in a poor observation environment.

## 5. Conclusions

We collected pseudorange observations from the rooftop of a building with only one reflecting surface in one specific direction. Other than that, it can be considered an open-sky environment. We calculated the speed, or range rate, from the recorded pseudorange measurements. Additionally, we computed range acceleration, the second time derivative of pseudoranges, and analyzed their characteristics. For an LoS signal, the range rate and range acceleration varied quite smoothly. However, the pseudorange fluctuations affected by a multipath signal were very high and unpredictable. We found that the range rate was inadequate for use as a weight because it varied by up to ±800 m/s, making it impossible to select a weight value corresponding to a specific range rate. On the other hand, for range acceleration, the LoS signal had a value near 0 m/s^2^, while the multipath signal displayed significant variability. Therefore, we tried a couple of models using range acceleration as a weight and found the exponential function model to be the most effective.

The use of pseudorange acceleration as a weight, in the form of an exponential function, significantly improved positioning accuracy. However, there were instances where the newly developed model was not effective. Upon further analysis, it was discovered that multipath signals could have low acceleration and be assigned a higher weight. We implemented a threshold for the SNR to address this issue. The new weight model was applied to measurement epochs with an SNR exceeding a predetermined upper limit. For epochs with a lower SNR, the range acceleration was set to a high value of 100 m/s^2^. This approach ensured consistent positioning accuracy throughout the observation period. The fusion strategy, which involves assigning weights based on range acceleration and a threshold SNR value, resulted in an 86% increase in 3D positioning accuracy compared to the no-weight case.

## Data Availability

The data should be available upon request to the corresponding author.
